# Editorial: Listening with two ears – new insights and perspectives in binaural research

**DOI:** 10.3389/fnins.2023.1323330

**Published:** 2023-11-07

**Authors:** Huiming Zhang, Yi Zhou, Lina Reiss

**Affiliations:** ^1^Department of Biomedical Sciences, University of Windsor, Windsor, ON, Canada; ^2^College of Health Solutions, Arizona State University, Tempe, AZ, United States; ^3^Department of Otolaryngology, Oregon Health and Science University, Portland, OR, United States

**Keywords:** binaural integration, sound localization, spatial release from masking, binaural fusion, auditory plasticity, auditory scene analysis, hearing loss, cochlear implant

While advantages of seeing with two eyes (i.e., binocular vision) were noted many centuries ago by ancient Greek scholars including Klaudios Ptolemaios (c. 100–c. 178CE), those of hearing with two ears (i.e., binaural hearing) were not reported until the end of the 18th century (Wells, 1792; Venturi, 1796, 1802). Great strides were made in the study of binaural hearing after the “Duplex Theory” of sound localization (Strutt, 1907), i.e., the involvement of both the interaural-level and the interaural-time difference (ILD and ITD), was established at the beginning of the last century. Major discoveries provided insight into some important aspects of binaural hearing including neural bases of sound localization (e.g., Jeffress, 1948; Goldberg and Brown, 1969; Colburn and Durlach, 1978; Durlach and Colburn, 1978; Moiseff and Konishi, 1981; Yin and Chan, 1990; Blauert, 1996). These early studies paved the way for addressing a wide range of questions related to functions and mechanisms of binaural hearing. Among these questions is how spatial cues can be used to aid in the detection of a sound in a noisy environment. Other important questions include how speech perception is dependent on the integration of temporal and spectral acoustic information received by the two ears, and how binaural hearing can be shaped by auditory experience. Recently, significant progress has been made in understanding disorders in binaural hearing, i.e., abnormal conditions related to alterations of central binaural integration rather than peripheral cochlear damage. Some of these latest findings are highlighted in the eighteen original research articles published on the present Research Topic.

## 1. Preview of studies on the present Research Topic

### 1.1. Binaural hearing in normal systems: spatial release from masking

One benefit of binaural hearing is that it aids in the recognition of a sound. In a natural acoustic environment, the detection and perception of a sound of interest can be masked by a background noise (Gelfand, 2004). A spatial separation between the two sounds can reduce the effect of masking, resulting in spatial release from masking (SRM) (Plomp and Mimpen, 1981; Saberi et al., 1991). A related phenomenon is the binaural masking-level difference (BMLD), in which the detection of a sound is improved when the phases of the sound at the two ears become different from those of a masker (Licklider, 1948).

SRM and BMLD were investigated in five studies in this Research Topic. Asim et al. demonstrated in the rat midbrain that neurophysiological responses of an ensemble of neurons to a sound could be suppressed by a preceding sound and the effect was only mildly dependent on local excitation/inhibition. Such a suppressive effect could be reduced by a spatial separation between the sounds, which was reminiscent of SRM. Fan et al. measured responses to diotic and dichotic tone-in-noise stimuli from individual neurons in the midbrain of awake rabbits and revealed that BMLD was related more to interaural correlation between sounds at the two ears than to ITD or ILD. Using a modeling/simulation approach, Smith et al. trained an artificial neural network to yield a BMLD performance that matched the performance of human listeners. Functions of inner nodes of the model resembled interaural correlation functions observed in animal neurophysiological studies, suggesting that BMLD is dependent on interaural correlation.

Previous investigations of masking and SRM have been conducted only under anechoic conditions and have not considered stimulus statistics. Biberger and Ewert extended such investigations to more complex environments by examining how factors such as room reverberation affected detection and quality perception of a target sound in the presence of colocalized or spatially separated maskers. Encke and Dietz characterized the interaural statistics of tone-in-noise stimuli, providing a basis for future studies of the relationship between these statistics and SRM.

### 1.2. Sound localization in abnormal/disordered systems

Understanding how sound localization is affected by hearing loss and other disorders can not only help develop clinical approaches to deal with such problems, but also provide insights into mechanisms underlying normal binaural hearing. Four studies in this Research Topic examined how sound localization was affected by aging, stroke, tinnitus, and replacement of natural acoustic stimulation by electrical stimulation generated by cochlear implants (CIs).

Previous studies have reported worsening of sound localization abilities in aging populations (see Russell, 2022 for review). In this Research Topic, Eddins et al. used electroencephalography to demonstrate that the processing of ITD was more heavily dependent on the activation of the contralateral than the ipsilateral auditory cortex. This asymmetry along with across-hemisphere differences in response waveform over specific time windows was reduced with age, which may be among the factors affecting the sensitivity to ITD in older adults. Dietze et al. found that lesions of specific brain regions caused by ischemic stroke impaired sound lateralization, with the impairment manifested in different ways depending on lesion sites. Specifically, brainstem lesions caused compressed and distorted response choices in lateralization, thalamic lesions led to a shift of perceived auditory space, and cortical lesions resulted in strong effects on lateralization of stimuli contralateral to the lesion. Long et al.s' study on sound-localization abilities in listeners with tinnitus showed that tinnitus percepts could affect localization of tones but not words. Future work is needed to determine the structure(s) within the auditory pathway that is/are responsible for such interference.

The acuity of sound-source localization, especially that based on ITD cues, is known to be significantly reduced in individuals with bilateral CIs (see Laback et al., 2015 for review). Müller et al. investigated this phenomenon using neurophysiological recordings and mathematical modeling/simulation. They revealed that sensitivities of neurons in the lateral superior olivary nucleus (LSO) to ITD were dependent on the temporal precision of spiking of inputs to the LSO from lower brainstem structures. In comparison to neural inputs to the LSO driven by acoustic stimulation, those driven by electrical stimulation (e.g., generated by CIs) exhibited hyper precision and low jitter, which led to reduced sensitivity to ITD in olivary neurons. This finding suggests that localization ability based on ITD can be improved by introducing jitter into stimulation generated by CIs.

### 1.3. Dependence of speech perception on binaural integration in normal and impaired auditory systems

A notable gap in literature exists regarding how speech perception depends on the integration of acoustic (including spectral) cues received by the two ears. Six studies in this Research Topic investigated effects of perturbation of this integration on speech perception.

Two studies used simulation to create asymmetries of inputs in normal-hearing listeners. Yoon and Morgan revealed that consonant recognition was possible even if large amounts of spectral information were missing at individual ears, as long as complementary information could be integrated across ears. This finding suggests that effective bimodal hearing (i.e., with one ear having a CI while the contralateral ear having acoustic hearing) can be achieved when the implanted ear is provided with information within a frequency range that complements rather than overlaps that of the contralateral ear. Anderson et al. used a vocoder to simulate CI processing and manipulate the dynamic range of speech at each ear to create a “better ear” and a “poorer ear”. Decreasing the dynamic range in one ear led to increased binaural interference for single words, whereas for dichotic double word presentations, this manipulation led to increased word fusion and blending. These findings suggest that increased binaural fusion due to dynamic range asymmetry can result in abnormal fusion and interference.

Abnormal fusion does occur in listeners with hearing loss and can lead to binaural interference as well as difficulties with speech understanding in a noisy environment. Oh et al. demonstrated in listeners with hearing aids that there was significant inter-subject variation in binaural pitch fusion, i.e., fusion of sounds with different pitches across ears. Broad binaural fusion was correlated with a reduced ability to use voice fundamental frequency differences in speech recognition in the presence of background talkers. This correlation was also observed in normal-hearing listeners, suggesting that underlying mechanisms are of central rather than peripheral origin. Burg et al. examined listening effort in users of bilateral CIs. They found that listening effort increased when a poorer ear was used in addition to a better ear, suggesting negative consequences of binaural integration when asymmetries in hearing are present between ears.

Two other studies developed new methods with potential application for future studies of binaural integration. Dolhopiatenko and Nogueira demonstrated that decoding of selective auditory attention could be obtained in bimodal CI users using electroencephalography signals, despite the presence of stimulus artifacts from the CI in these signals. Chou et al. developed an algorithm based on a biologically inspired network to process both special and directional acoustic information driven by the two ears. This algorithm is able to segregate sounds based on spatial and spectral information and may also have applications in the development of hearing devices or software. Methods used in both studies provide researchers an opportunity to explore how binaural integration contributes to neural processing.

### 1.4. Brain plasticity: auditory training and the influence of auditory experience

Another emerging area of research is about how binaural hearing and underlying mechanisms are shaped by auditory experience. Nisha et al. showed in listeners with hearing loss that auditory training using stimuli delivered in a virtual acoustic space improved spatial acuity of sound localization. Ding et al. examined the detection of a binaural gap, i.e., a period without correlation between acoustic signals received by the two ears, in listeners with normal hearing. Performance was correlated with the sensitivity to temporal fine structure of monaural acoustic stimulation, and this correlation was reduced by musical training. Sanchez Jimenez et al. used the ferret as a model system to study plastic changes in sound-localization behaviors following unilateral conductive hearing loss. They found that training facilitated recovery of sound localization abilities. Recovery could generalize to more naturalistic listening conditions, so long as the target sounds provided sufficient spatial information.

## 2. Significance and future directions

The current Research Topic explored some exciting directions in the field of binaural hearing using both normal and disordered/clinically relevant systems. These studies provide new knowledge about functions and underlying mechanisms of some established binaural phenomena. They also show how binaural hearing can be shaped by auditory experience and provide new applications of electrophysiological tools and computational models. Despite these advances, many important questions remain to be answered. For instance, how does the brain use spatial along with temporal and spectral cues to stream and group information to form cohesive individual acoustic images? Conversely, how is this information used to segregate multiple acoustic images, as in the cocktail party effect? A multidisciplinary approach is needed to address these questions and help understand how the auditory scene is analyzed by the brain. Human psychoacoustical and animal behavioral experiments can improve our understanding of binaural hearing at the functional level. Neurophysiological recordings along with neurostimulation, and neuropharmcological or molecular/genetic manipulation conducted in normal and disordered systems may reveal key binaural components through gain-of-function and loss-of-function analyses. Mathematical models will be critical for simulating binaural components not easily measured/manipulated using experimental techniques. Taken together, multiple approaches integrated across studies as well as within studies will pave the way for future advances in the study of binaural hearing.

## Author contributions

HZ: Conceptualization, Writing—original draft, Writing—review & editing. YZ: Conceptualization, Writing—original draft, Writing—review & editing. LR: Conceptualization, Writing—original draft, Writing—review & editing.
